# Characteristics, Perceived Side Effects and Benefits of Electronic Cigarette Use: A Worldwide Survey of More than 19,000 Consumers

**DOI:** 10.3390/ijerph110404356

**Published:** 2014-04-22

**Authors:** Konstantinos E. Farsalinos, Giorgio Romagna, Dimitris Tsiapras, Stamatis Kyrzopoulos, Vassilis Voudris

**Affiliations:** 1 Onassis Cardiac Surgery Center, Sygrou 356, Kallithea 17674, Greece; E-Mails: dtsiapras@hotmail.com (D.T.); stkyrz@gmail.com (S.K.); vvoudris@otenet.gr (V.V.); 2 ABICH S.r.l, Biological and Chemical Toxicology Research Institute, Via 42 Martiri, 213, Verbania (VB) B-28924, Italy; E-Mail: giorgio.romagna@gmail.com

**Keywords:** lectronic cigarette, smoking, tobacco, nicotine, harm reduction, public health

## Abstract

*Background*: Electronic cigarette (EC) use has grown exponentially over the past few years. The purpose of this survey was to assess the characteristics and experiences of a large sample of EC users and examine the differences between those who partially and completely substituted smoking with EC use. *Methods*: A questionnaire was prepared, translated into 10 different languages and uploaded in an online survey tool. EC users were asked to participate irrespective of their current smoking status. Participants were divided according to their smoking status at the time of participation in two subgroups: former smokers and current smokers. *Results*: In total, 19,414 participants were included in the analysis, with 88 of them (0.5%) reported not being smokers at the time of EC use initiation. Complete substitution of smoking was reported by 81.0% of participants (former smokers) while current smokers had reduced smoking consumption from 20 to 4 cigarettes per day. They were using ECs for a median of 10 months. They initiated EC use with a median of 18 mg/mL nicotine-concentration liquids; 21.5% used higher than 20 mg/mL. Only 3.5% of participants were using 0-nicotine liquids at the time of the survey. Former smokers were highly dependent (Fagerström Test for Cigarette Dependence = 7) and were heavier smokers (21 cigarettes per day when smoking) compared to current smokers. The most important reasons for initiating EC use for both subgroups was to reduce the harm associated with smoking and to reduce exposure of family members to second-hand smoking. Most considered ECs as less harmful than tobacco cigarettes, while 11.0% considered them absolutely harmless. Side effects were reported by more than half of the participants (59.8%), with the most common being sore/dry mouth and throat; side effects were mild and in most cases were subsequently resolved (partially or completely). Participants experienced significant benefits in physical status and improvements in pre-existing disease conditions (including respiratory disease such as asthma and chronic obstructive lung disease). Being former smoker was independently associated with positive effects in health and improvements in disease conditions. *Conclusions*: The results of this worldwide survey of dedicated users indicate that ECs are mostly used to avoid the harm associated with smoking. They can be effective even in highly-dependent smokers and are used as long-term substitutes for smoking. High levels of nicotine are used at initiation; subsequently, users try to reduce nicotine consumption, with only a small minority using non-nicotine liquids. Side effects are minor and health benefits are substantial, especially for those who completely substitute smoking with EC use. Further population and interventional studies are warranted.

## 1. Introduction

Electronic cigarettes (ECs) have been marketed in recent years as alternatives to smoking. They are battery-operated devices, used to vaporise a liquid that may or may not contain nicotine. The main ingredients of liquids are propylene glycol, glycerol and several flavourings. A large variety of devices are available, from small cigarette-like devices consisting of a low-capacity disposable lithium battery and a prefilled atomiser (commonly called cartomiser) to new-generation high-capacity rechargeable batteries that can deliver adjustable voltage and atomisers that are able to store more liquid and can be refilled. 

Currently-approved methods for smoking cessation have low long-term quit rates. Nicotine replacement therapies have less than 7% sustained abstinence rate [1], while oral medications have less that 20% quit rate at one year [2]. Therefore, tobacco harm reduction strategies and products have been developed, with the goal to reduce smoking-related disease burden by providing nicotine in a less harmful form [3]. ECs are tobacco harm reduction products that may deal with both chemical (through nicotine delivery) and behavioural (through motor simulation and sensory stimulation) addiction to smoking [4]. Awareness and use of ECs are growing exponentially. Surveys have shown that they may be effective in promoting reduction of cigarette consumption or even complete abstinence [5,6]. Cross-sectional studies have raised doubts whether ECs promote smoking cessation [7,8] but two recently-published randomized studies showed a small but significant potential of ECs to promote smoking reduction and cessation [9,10]. However, organisations like the World Health Organisation and the US Food and Drug Administration prohibit the declaration of any therapeutic claims. Additionally, there is controversy on the nicotine-delivery potential of ECs. Some studies have shown that, despite being effective at suppressing smoking withdrawal symptoms, there was minimal nicotine absorption [11]. More recently, studies on experienced users have shown that they have elevated salivary cotinine levels [12], while the use of modern devices resulted in significant nicotine absorption [13]. 

In order to better understand the characteristics of ECs use and the perceived benefits or negative experience, internet surveys have been conducted [5,6,14]. Some of them included a limited number of participants while most questionnaires were available in one or two languages, which restricted international participation and sharing of experiences. Therefore, the purpose of this study was to assess the characteristics of a worldwide sample of EC users, by providing a questionnaire in several languages and by promoting the study through the internet. Since ECs are used by consumers as either partial or complete substitutes for smoking, we sought to examine the differences in characteristics, patterns of use, benefits and side effects between these two subgroups.

## 2. Methods

A questionnaire was developed and uploaded in an online survey tool (www.surveymonkey.com). The questionnaire was available in 10 languages (Czech, English, French, German, Greek, Hungarian, Italian, Polish, Russian, and Spanish). At least two native speakers (one of whom was a qualified translator) checked the validity of each translation, based on the original English questionnaire. A brief presentation of the research purpose and informed consent in each language was uploaded in a website of EC advocates (www.ecigarette-research.com). Participants had to push the “I agree” button in order to be transferred to the respective questionnaire. Data were collected from April 2013 until July 2013. The survey was approved by the ethics committee of our institution. 

Participants were aged >18 years; current, former or never smokers were eligible. Their IP addresses were recorded in order to identify and delete duplicate records. They could provide an email address to be contacted regarding future studies. The questionnaire had three main sections, asking for information about: (1) baseline characteristics of the participants, including age, gender, education, and country of residence. (2) Past and current smoking status and EC patterns of use. Assessment of smoking dependence was performed by using the Fagerström Test for Cigarette Dependence (FTCD) [15]. Included were questions to assess participants’ opinion about the risk profile of ECs and reasons for initiating EC use. The latter was assessed by asking participants to provide a score from 1 (not important) to 5 (most important) for all answer options. Moreover, they were asked to report how many times they had attempted to quit smoking in the past. (3) Health-related issues. Participants were asked to report benefits and side effects that they experienced after initiation of EC use. Included were questions about accidents related to EC use. Additionally, there were questions about the progression of previously-established disease conditions.

## 3. Statistical Analysis

The sample was divided into current and former-smokers, according to their reported status at the time of participation in the survey. Results are reported for the whole sample and for each of the subgroups. Participants who reported initiating ECs without being smokers were evaluated separately. The sample size varied by variable because of missing data; therefore, for some questions, the sum of responses may be less than 100%. In some questions, responders were allowed to choose more than one option; in these cases, each answer is presented separately and the sum of responses may exceed 100%. Continuous variables are reported as median (interquartile range (IQR)), because medians are less sensitive to extreme values. Categorical variables are reported as number (percentage). Mann Whitney U test was used to compare continuous variables between current and ex-smokers, while cross tabulations with χ^2^ test were used for categorical variables. Wilcoxon signed ranks test was used to compare cigarette consumption before and after initiation of EC use in current smokers. To assess whether being a former smoker was independently associated with improvements in disease conditions and positive experience in physiologic functions, stepwise binary logistic regression analyses were performed. A separate analysis was performed for each variable with the condition being the dependent variable; physiologic conditions were encoded as no change/worse *vs*. better. Included as covariates were being former/current smoker, age, gender, education, the answer to the question “Where did you hear about this survey” (encoded as internet users’ forums *vs*. other), smoking duration, daily cigarette consumption before EC use initiation, EC duration of use, EC consumption and nicotine content in ECs. All analyses were performed with commercially available software (SPSS ver. 18, Chicago, IL, USA).

## 4. Results

### 4.1. Participant Characteristics

After excluding double IP addresses (including those answering more than one translation of the questionnaire), 19,441 participants were included in the analysis with 88 (0.5%) mentioning that they were not smokers while 19,353 reported being smokers before initiating EC use. The baseline characteristics of the latter population are displayed in Table 1. More than one-third of participants used the English translation of the questionnaire. Distribution of responders by region of residence was: 74.7% from Europe, 20.7% from America, 1.8% from Asia, 1.1% from Australia, and 0.2% from Africa. The median age of the participants was 39 years, with significantly higher proportion being males. Almost half of them received higher education. From the whole sample, 81.0% reported that they had completely quit smoking at the time of participation to the survey. Former smokers were older, with higher male prevalence and higher education level compared to current smokers. Most participants were informed about this survey from EC users’ internet forums.

### 4.2. Past and Current Smoking Status—EC Use Patterns and Beliefs

Past and current smoking status and EC use patterns are displayed in Table 2. Former smokers were smoking for longer duration and had higher daily cigarette consumption. Almost one-third of current smokers were smoking occasionally (less than daily) while the rest mentioned that their daily consumption was reduced from 20 to 4 cigarettes per day. The FTCD for the whole population was 7 (5–9), with former smokers having higher score compared to current smokers. Former smokers also reported more attempts to quit smoking in the past. The median duration of EC use for the whole group was 10 months, with 97.1% using it on a daily basis. The majority of vapers were using second- (eGo-type) and newer-generation (also called “Mods”) devices while only 3.7% reported using cigarette-like devices. More than one-third was using “do-it-yourself” liquids (buying base ingredients and concentrated flavours which they subsequently mix). A reduction in nicotine levels used in EC liquid was observed as time of use progressed, from a median level of 18 mg/mL at initiation of use to 12 mg/mL at the time of participation to the survey. Nicotine concentration of more than 20 mg/mL was the initial choice for 21.5% of the population, with former smokers being more likely to use such nicotine levels (23.3% *vs*. 13.8% for current smokers, χ^2^ = 155.9, *p* < 0.001). Only 3.5% of the participants were using 0-nicotine liquids at the time of the survey. For former smokers, the median time to quit smoking since initiation of EC use was 1 month. 

**Table 1:** Baseline characteristics of participants *.

Characteristic	Total (n = 19,353)	Current smokers (n = 3682)	Former smokers (n = 15,671)	Statistic	*p* value
Participants	19,353	3682 (19.0)	15,671 (81.0)		
Translation					
Czech	237 (1.2)	53 (1.4)	184 (1.2)	χ^2^ = 459.1	<0.001
English	6803 (35.2)	823 (22.4)	5980 (38.2)		
French	2225 (11.5)	617 (16.8)	1608 (10.3)		
German	3974 (20.5)	796 (21.6)	3178 (20.3)		
Greek	783 (4)	204 (5.5)	579 (3.7)		
Hungarian	470 (2.4)	79 (2.1)	391 (2.5)		
Italian	2451 (12.7)	637 (17.3)	1814 (11.6)		
Polish	1153 (6)	265 (7.2)	888 (5.7)		
Russian	887 (4.6)	124 (3.4)	763 (4.9)		
Spanish	370 (1.9)	84 (2.3)	370 (1.9)		
Where did you hear about this survey?					
EC users' forums	14,097 (72.8)	2427 (65.9)	11,670 (74.5)	χ^2^ = 125.2	<0.001
Internet search engines	1880 (9.7)	395 (10.7)	1485 (9.5)		
Family/friends	1030 (5.3)	263 (7.1)	767 (4.9)		
Physical/internet EC shops	2223 (11.5)	567 (15.4)	1656 (10.6)		
TV/Radio/Newspapers	73 (0.4)	18 (0.5)	55 (0.4)		
Age (years)	39 (31–47)	38 (30–46)	39 (32–47)	U = 26,144,506	<0.001
Gender (male)	14,544 (76.3)	2686 (72.9)	11,858 (75.7)	χ^2^ = 12.3	<0.001
Education					
Less than high school	1980 (10.3)	419 (11.4)	1561 (10.0)	χ^2^ = 11.0	0.004
High school	7995 (41.5)	1552 (42.4)	6443 (41.3)		
Higher education	9294 (48.2)	1689 (46.1)	7605 (48.7)		

Abbreviations: EC, electronic cigarettes. * Excluding participants who reported that they were non-smokers before EC use initiation.

**Table 2:** Past and current smoking status and electronic cigarette use patterns.

Characteristic	Total (n = 19,353)	Current smokers (n = 3682)	Former smokers (n = 15,671)	Statistic	*p* value
Smoking history					
Years smoking	20 (14–30)	20 (12–30)	20 (14–30)	U = 26,489,867	<0.001
Cigarettes per day	20 (18–30)	20 (16–30)	21 (18–30)	U = 26,909,388	<0.001
FTCD	7 (5–8)	6 (5–8)	7 (5–8)	U = 26,341,216	<0.001
Total past quit attempts	3 (0–6)	2 (0–5)	3 (0–7)	U = 24,851,864	<0.001
Current smokers’ status					
Daily smokers		2521 (68.5)			
Occasional smokers		1132 (30.7)			
Cigarettes per day now		4 (2–7)		Z = −42.1 ^1^	<0.001
EC duration of use	10 (4–19)	8 (4–17)	11 (5-19)	U = 26,287,110	<0.001
EC use pattern					
Daily	18,784 (97.1)	3466 (94.2)	15,318 (97.7)	χ^2^ = 161.5	<0.001
Occasionally	432 (2.2)	184 (5.0)	248 (1.6)		
Not anymore	122 (0.6)	29 (0.8)	93 (0.6)		
EC device most often used					
Cigarette-like	715 (3.7)	219 (5.9)	485 (3.1)	χ^2^ = 294.2	<0.001
eGo batteries	8214 (42.7)	1912 (51.9)	6266 (40.0)		
“Mods”	10,329 (53.6)	1511 (41.0)	8785 (56.1)		
EC liquid use	19,353				
prefilled cartomisers	305 (1.6)	99 (2.7)	206 (1.3)	χ^2^ = 76.0	<0.001
ready-to-use liquids	11,638 (60.4)	2357 (64.0)	9281 (59.2)		
do-it-yourself liquids	7315 (38.0)	1211 (32.9)	6104 (39)		
EC daily consumption					
mL liquid per day	3 (2–5)	3 (2–4)	3 (2–5)	U = 23,003,765	<0.001
nr of cartridges per day	1 (1–3)	1 (1–2)	2 (1–3)	U = 8893	0.066
Current nicotine levels in EC	12 (7–16)	12 (8–16)	12 (6–16)	U = 27,681,658	0.004
Nicotine levels at initiation of EC use	18 (12–18)	18 (11–18)	18 (12–19)	U = 23,196,983	<0.001

Abbreviations. FTCD, Fagerström Test for Cigarette Dependence; EC, electronic cigarette. ^1^ Comparison with cigarette consumption before initiation of EC use (Wilcoxon signed rank test).

Examining the reasons for initiating EC use (Table 3), reducing or quitting smoking because it is not a healthy habit had the highest score (median = 5, IQR: 4–5). Reducing secondary smoking exposure to family members was scored as a very important reason, while lower scores were given to economic reasons, enjoying flavours variability and avoiding smoking ban in public places.

The vast majority of participants reported that they considered ECs less harmful than tobacco. Less than 1% considered them equally or more harmful than tobacco; 11.0% considered ECs completely harmless, with former smokers being more likely to report this.

**Table 3:** Reasons for electronic cigarette use initiation and concepts about their risk profile.

Reason	Total (n = 19,353)	Current smokers (n = 3682)	Former smokers (n = 15,671)	Statistic	*p* value
Reasons for initiating EC use ^1,2^					
Reduce/quit smoking because it is not a healthy habit	5 (4–5)	8839.9	9799.9	U = 25,628,315	<0.001
Reduce smoking exposure to family members	4 (3–5)	8546.5	9673.2	U = 24,226,257	<0.001
Avoid smoking ban in public places	2 (1–3)	10,024.5	9234.1	U = 24,783,083	<0.001
Economic reasons (ECs cheaper)	3 (2–4)	10,100.4	9337.3	U = 25,478,230	<0.001
Enjoy the variability of flavours in ECs	3 (2–4)	9550.8	9348.6	U = 26,529,912	0.040
Compared to tobacco, ECs are:					
Absolutely harmless	2124 (11.0)	313 (8.5)	1811 (11.6)	χ^2^ = 86.6	<0.001
Less harmful than tobacco cigarettes	17,063 (88.2)	3300 (89.6)	13,763 (87.8)		
Equally harmful to tobacco cigarettes	97 (0.5)	46 (1.2)	51 (0.3)		
More harmful than tobacco cigarettes	27 (0.1)	12 (0.3)	15 (0.1)		

Abbreviations. EC, electronic cigarette. ^1 ^Participants were asked to provide a score from 1 (not important) to 5 (most important) for each answer option. ^2^ Median (interquartile range) reported for the whole sample, median rank reported for each group (Mann-Whitney test).

### 4.3. Health-Related Issues

More than half of the participants (57.9%) reported at least one adverse symptom that they attributed to EC use (Table 4). The most commonly reported symptom was sore or dry mouth and throat (38.9%). Cough and gum problems were reported by a smaller proportion (12.8% and 13.1% respectively); the former was more common in current smokers while the latter was more prevalent in former smokers. More than 90% reported complete or partial resolution of the symptoms. Resolution was positively associated with duration of EC use (linear regression analysis: β = 0.114, *p* < 0.001).

**Table 4:** Side effects and accidents associated with electronic cigarette use.

Side effects/accidents ^1^	Total (n = 19,353)	Current smokers (n = 3682)	Former smokers (n = 15,671)	Statistic	*p* value
Sore or dry mouth and throat	7520 (38.9)	1441 (39.1)	6079 (38.8)	χ^2^ = 0.1	0.699
Headache	2140 (11.1)	433 (11.8)	1707 (10.9)	χ^2^ = 2.3	0.131
Gingivitis/gum bleeding	2534 (13.1)	273 (7.4)	2261 (14.4)	χ^2^ = 128.8	<0.001
Mouth or tongue sores/inflammation	973 (5.0)	151 (4.1)	822 (5.2)	χ^2^ = 8.2	0.004
Black tongue	145 (0.7)	31 (0.8)	114 (0.7)	χ^2^ = 0.5	0.469
Nose bleeding	601 (3.1)	84 (2.3)	517 (3.3)	χ^2^ = 10.3	0.001
Cough	2475 (12.8)	556 (15.1)	1919 (12.2)	χ^2^ = 21.8	<0.001
Dizziness	991 (5.1)	196 (5.3)	795 (5.1)	χ^2^ = 0.4	0.536
Sleepiness	661 (3.4)	139 (3.8)	522 (3.3)	χ^2^ = 1.8	0.182
Sleeplessness	1211 (6.3)	202 (5.5)	1009 (6.4)	χ^2^ = 4.6	0.032
Heart palpitations	959 (5.0)	216 (5.9)	743 (4.7)	χ^2^ = 8.0	0.005
Breathing difficulties	395 (2.0)	91 (2.5)	304 (1.9)	χ^2^ = 4.2	0.040
Allergies	343 (1.8)	57 (1.5)	286 (1.8)	χ^2^ = 1.3	0.252
Chest pain	613 (3.2)	142 (3.9)	471 (3.0)	χ^2^ = 7.0	0.008
No side effects	7789 (40.2)	1478 (40.1)	6311 (40.3)	χ^2^ = 0.0	0.884
Did the above-mentioned symptoms resolve over time? ^2^					
				
Completely resolved	6873 (59.9)	1122 (51.6)	5751 (61.8)	χ^2^ = 87.1	<0.001
Partially resolved	3968 (34.6)	877 (40.4)	3091 (33.2)		
Completely unresolved	631 (5.5)	174 (8.0)	457 (4.9)		
Accidents associated with EC use					
Associated with EC liquid	294 (1.5)	77 (2.1)	217 (1.4)	χ^2^ = 9.9	0.002
Associated with the battery	180 (0.9)	50 (1.4)	130 (0.8)	χ^2^ = 9.0	0.003
Associated with other electrical parts	125 (0.6)	36 (1.0)	89 (0.6)	χ^2^ = 7.8	0.005

Data presented as number (percent) or median (interquartile range). ^1 ^Participants were allowed to choose more than one option. ^2^ Percent of those who reported the respective side effects.

A small minority reported accidents associated with EC use. Most common was accidental exposure to EC liquid (1.5% of participants), with lower prevalence for accidents associated with batteries and other electrical parts.

More than half of the participants reported better breathing, olfactory and gustatory senses, endurance and physical status in general after initiation of EC use (Table 5). More than one-third reported better quality of sleep, while smaller proportions mentioned improvements in mood, appetite and sexual performance. In all cases, former smokers reported more beneficial effects compared to current smokers.

**Table 5:** Changes in physiologic functions after electronic cigarette use initiation.

Changes	Total (n = 19,353)	Current smokers (n = 3682)	Former smokers (n = 15,671)	Statistic	*p* value
After initiating EC use, have you experienced any changes in:					
Physical status in general					
Worse	79 (0.4)	24 (0.7)	55 (0.4)	χ^2^ = 308.6	<0.001
No change	4769 (24.6)	1309 (35.6)	3460 (22.1)		
Better	14,409 (74.5)	2316 (62.9)	12,093 (77.2)		
Smell					
Worse	29 (0.1)	12 (0.3)	17 (0.1)	χ^2^ = 518.4	<0.001
No change	2538 (13.1)	894 (24.3)	1644 (10.5)		
Better	16,722 (86.4)	2743 (74.5)	13,979 (89.2)		
Taste					
Worse	62 (0.3)	26 (0.7)	36 (0.2)	χ^2^ = 431.6	<0.001
No change	3359 (17.4)	1051 (28.5)	2308 (14.7)		
Better	15,857 (81.9)	2572 (69.9)	13,285 (84.8)		
Breathing					
Worse	137 (0.7)	40 (1.1)	97 (0.6)	χ^2^ = 304.0	<0.001
No change	2497 (12.9)	784 (21.3)	1713 (10.9)		
Better	16,641 (86.0)	2824 (76.7)	13,817 (88.2)		
Appetite					
Worse	218 (1.1)	56 (1.5)	162 (1.0)	χ^2^ = 41.5	<0.001
No change	12,807 (66.2)	2564 (69.6)	10,243 (65.49)		
Better	6216 (32.1)	1022 (27.8)	5194 (33.1)		
Sexual performance					
Worse	87 (0.4)	22 (0.6)	65 (0.4)	χ^2^ = 88.4	<0.001
No change	13,844 (71.5)	2838 (77.1)	11,006 (70.2)		
Better	5303 (27.4)	776 (21.1)	4527 (28.9)		
Mood					
Worse	576 (3.0)	155 (4.2)	421 (2.7)	χ^2^ = 158.8	<0.001
No change	12,478 (64.5)	2622 (71.2)	9856 (62.9)		
Better	6207 (32.1)	867 (23.5)	5340 (34.1)		
Memory					
Worse	242 (1.3)	68 (1.8)	174 (1.1)	χ^2^ = 65.0	<0.001
No change	15,868 (82.0)	3124 (84.8)	12,774 (81.5)		
Better	3128 (16.2)	444 (12.1)	2684 (17.1)		
Quality of sleep					
Worse	651 (3.4)	153 (4.2)	498 (3.2)	χ^2^ = 121.7	<0.001
No change	11,224 (58.0)	2383 (64.7)	8841 (56.4)		
Better	7372 (38.1)	1106 (30.0)	6266 (40.0)		
Endurance					
Worse	84 (0.4)	31 (0.8)	53 (0.3)	χ^2^ = 294.0	<0.001
No change	4945 (25.6)	1326 (36.0)	3619 (23.1)		
Better	14,231 (73.5)	2287 (62.1)	11,944 (76.2)		

Abbreviations. EC, electronic cigarette.

Participants were additionally asked whether they suffered from any chronic health conditions before initiating EC use. Options included: diabetes, hypertension, hypercholesterolemia, thyroid disease, coronary artery disease (CAD), asthma, chronic obstructive lung disease (COPD). In total, 5259 reported suffering from a chronic disease. The answers are displayed in Table 6. The highest prevalence was reported for hypertension, followed by hypercholesterolemia, asthma and COPD. In all disease conditions, higher proportions of former smokers compared to current smokers reported that their condition improved after switching to EC use; the highest proportion reporting improvement were participants with COPD. Worsening of their condition was reported by 0.9% of people with diabetes, 0.8% with hypertension, 0.9% with hypercholesterolemia, 1.6% with thyroid disease, 2.2% with CAD, 1.1% with asthma and 0.8% with COPD. 

**Table 6:** Changes in disease conditions after electronic cigarette use initiation.

Side effects/accidents	Total (n = 19,353)	Current smokersl (n = 3682)	Former smokersl (n = 15,671)	Statistic		*P* value
Did you suffer from any of these conditions before initiating EC use? ^1^						
Diabetes	574 (3.0)	90 (2.4)	484 (3.1)	χ^2^ = 4.3		0.038
Hypertension	2365 (12.2)	390 (10.6)	1975 (12.6)	χ^2^ = 11.2		0.001
Hypercholesterolemia	1580 (8.2)	292 (7.9)	1288 (8.2)	χ^2^ = 0.3		0.565
Thyroid disease	622 (3.2)	127 (3.4)	495 (3.2)	χ^2^ = 0.8		0.368
Coronary artery disease	318 (1.6)	68 (1.8)	250 (1.6)	χ^2^ = 1.2		0.280
Asthma	1308 (6.8)	227 (6.2)	1081 (6.9)	χ^2^ = 2.5		0.111
COPD	1190 (6.1)	230 (6.2)	960 (6.1)	χ^2^ = 0.1		0.784
Did you experience any change in these conditions after initiating EC use? ^2^						
Diabetes						
Worse	5 (0.9)	2 (2.2)	3 (0.6)	χ^2^ = 7.0		0.030
Stable	309 (53.8)	58 (64.4)	251 (51.9)			
Improved	230 (40.1)	27 (30.0)	203 (41.9)			
Hypertension						
Worse	19 (0.8)	6 (1.5)	13 (0.7)	χ^2^ = 33.8	<0.001	
Stable	944 (39.9)	194 (49.7)	750 (38.0)			
Improved	1149 (49.9)	139 (35.6)	1040 (52.7)			
Hypercholesterolemia						
Worse	14 (0.9)	5 (1.7)	9 (0.7)	χ^2^ = 35.2	<0.001	
Stable	724 (45.8)	167 (57.2)	557 (43.2)			
Improved	666 (42.2)	77 (26.4)	589 (45.7)			
Thyroid disease						
Worse	10 (1.6)	3 (2.4)	7 (1.4)	χ^2^ = 9.2	0.010	
Stable	367 (59.0)	84 (66.1)	283 (57.2)			
Improved	218 (35.0)	30 (23.6)	188 (38.0)			
Coronary artery disease						
Worse	7 (2.2)	4 (5.9)	3 (1.2)	χ^2^ = 12.6	0.002	
Stable	116 (36.5)	30 (44.1)	86 (34.4)			
Improved	171 (53.8)	24 (35.3)	147 (58.8)			
Asthma						
Worse	14 (1.1)	5 (2.2)	9 (0.8)	χ^2^ = 27.3	<0.001	
Stable	303 (23.2)	78 (34.4)	225 (20.8)			
Improved	856 (65.4)	116 (51.1)	742 (68.6)			
COPD						
Worse	10 (0.8)	4 (1.7)	6 (0.6)	χ^2^ = 9.5	0.009	
Stable	151 (12.7)	39 (17.0)	112 (11.7)			
Improved	901 (75.7)	158 (68.7)	743 (77.4)			
For those with lung disease, did your physician alter the medications you regularly use?	2498	457	2041			
Dosage/number increased	17 (0.7)	7 (1.5)	10 (0.5)	χ^2^ = 35.5	<0.001	
Dosage/number similar	644 (25.8)	169 (37.0)	475 (23.3)			
Dosage/number decreased	459 (18.4)	82 (17.9)	377 (18.5)			
Medications stopped	460 (18.4)	60 (13.1)	400 (19.6)			

Abbreviations. EC, electronic cigarette; COPD, chronic obstructive pulmonary disease. ^1^ Participants were allowed to choose more than one option. ^2^ Percentage of those reporting that they suffered from the respective disease condition.

For patients with lung disease (asthma or COPD) an additional question was asked whether their physician altered their medications after initiating EC use; more than one-third of patients reported reduction in dosage/number or complete cessation of medications.

### 4.4. Multivariate Analysis

The results of multivariate analyses are displayed in Table 7. Being a former smoker was independently associated with higher odds of experiencing positive effects in physiologic functions. Moreover, this subgroup had higher odds of reporting improvement in the disease conditions asked, especially for hypertension, cholesterol levels and lung disease.

**Table 7:** Multivariate analyses to assess the association between being former smoker and experiencing positive effects in physiologic functions and improvement in disease conditions.

Dependent variable	Former smoker	
	OR (95% CI)	*p* value
Physiologic functions		
Physical status	1.85 (1.70–2.00)	<0.001
Smell	2.66 (2.41–2.93)	<0.001
Taste	2.31 (2.11–2.53)	<0.001
Breathing	2.10 (1.90–2.31)	<0.001
Appetite	1.28 (1.18–1.39)	<0.001
Sexual performance	1.52 (1.39–1.67)	<0.001
Mood	1.67 (1.53–1.82)	<0.001
Memory	1.49 (1.33–1.67)	<0.001
Sleep	1.53 (1.41–1.66)	<0.001
Endurance	1.90 (1.75–2.06)	<0.001
Disease conditions		
Diabetes	1.81 (1.07–3.07)	0.026
Hypertension	1.96 (1.52–2.53)	<0.001
Cholesterol	2.20 (1.61–3.02)	<0.001
Thyroid disease	1.80 (1.12–2.89)	0.015
CAD	2.02 (1.01–4.04)	0.048
Asthma	2.23 (1.58–3.15)	<0.001
COPD	1.73 (1.16–2.58)	0.008

Abbreviations: CAD, coronary artery disease; COPD, chronic obstructive lung disease.

### 4.5. EC-Use Initiation by Non-Smokers

As already mentioned, 88 of the participants (0.5%) reported that they were not smokers at the time of initiation of EC use. Interestingly, seven of them (8.0%) responded to the questions of the FTCD and 11 more (12.5%) reported past attempts to quit smoking, suggesting that these were former smokers who quit smoking before initiating EC use. More than half of the participants (58.0%) were residents of European countries, while the rest were from America (30.7%) and Asia (8.0%). University/college education was reported by 51.1% of this group. They were using the ECs for 4 (2–8) months; 69.3% were using ready-to-use liquids while 10.2% were using prefilled cartomizers. The median liquid consumption was 1 (1–3) mL and 1 (1–1) cartomizers, respectively. Nicotine levels used at the time of participation was 0 (0–9) mg, with 53.4% reporting using non-nicotine liquids. Nicotine levels at initiation of EC use were 0 (0–12) mg; still 52.3% reported initiating EC use with non-nicotine liquids. The vast majority (97.8%) answered that ECs are absolutely safe (33.0%) or less harmful that tobacco cigarettes (64.8%). None of them was smoking tobacco cigarettes at the time of participation to the survey.

Forty-eight (54.5%) reported at least one of the side-effects mentioned in Table 4. Most common side effects were sore/dry throat (22.7%), headache (11.4%), dizziness (9.1%) and sleeping disorders (11.1%). Less than 5% reported oral sores/inflammation, cough, chest pain, nose bleeding, palpitations and breathing difficulties. These symptoms were completely unresolved in 4.5% of the participants. Accidents associated with EC equipment happened in seven participants; one had accidental exposure to EC liquid, while four and two others mentioned accidents associated with the battery or other electrical equipment respectively.

From physiologic functions mentioned in Table 5, a small minority reported worsening of any condition. The highest prevalence was for worsening of appetite (5.7%). The majority of the participants reported no changes in any physiologic functions.

Sixteen of the participants (18.2%) reported suffering from conditions listed in Table 6, with 81.3% mentioning that the condition remained stable. Only one participant reported that his condition (asthma) got worse after EC use initiation. 

## 5. Discussion

This is the first worldwide survey of EC users with the questionnaire translated in multiple languages that would allow a significant number of users to participate without the barrier or misunderstandings associated with a foreign language. Participants were overwhelmingly smokers who tried ECs mainly as a substitute to avoid the adverse effects of smoking. A very small minority were subjects who were not smokers at the time of EC use initiation. The main results of this survey indicate that ECs may be an effective substitute for smoking even in highly dependent subjects who are heavy smokers. Significant benefits are experienced by these people in physiologic functions and in some disease conditions, with former smokers (those who completely substituted smoking with EC use) being more likely to report such beneficial effects. A substantial proportion reported side effects, which were generally mild and in most cases partially or completely resolved after the initial period of EC use.

It should be emphasized that participants in these surveys are mostly dedicated users. Herein, this is verified by the fact that the majority of subjects heard about this survey from EC users’ forums. It is expected that such a population has more positive experience from EC use. Other subgroups such as people who were using ECs in the past but are no longer using them, due to either failure to reduce smoking or negative experience and side effects, are not motivated to participate to such surveys. Therefore the results should be interpreted with caution and cannot be extrapolated to the general population. The 81% of participants reporting complete smoking substitution cannot be interpreted as the true potential of ECs in smoking cessation in the general population; controlled studies have found much lower cessation rates [9,10], although such studies cannot take into account the large variability of products available in the market which gives users the opportunity to choose devices based on personal preference. Still, surveys are valuable tools in understanding the population of EC users and the way they use ECs in order to reduce or completely substitute smoking. Participation from the Asian continent was low; that was expected to occur considering the languages that were available. It would be interesting to evaluate EC use in countries such as China and Japan where anti-smoking rules are less strict.

An interesting finding of this study was that former smokers were more dependent on smoking (based on the FTCD), had slightly higher cigarette consumption and had made more attempts in the past to quit smoking compared to current smokers. It is expected to be more difficult for this population to completely substitute smoking with EC use. The retrospective nature of assessing dependence could have biased the results. However, similar observations were made in previous studies [4,5], and such findings could at least indicate that ECs may be a feasible option as a smoking substitute even for highly dependent and heavy smokers.

Both former and current smokers initiated EC use with high nicotine-containing liquids. More than one-fifth of the population initiated use with more than 20 mg/mL nicotine concentration, with higher prevalence in former smokers, supporting the hypothesis that nicotine plays an important role in the success of ECs as smoking substitutes [4,16]. This can be attributed to the lower nicotine absorption from EC use compared to smoking [13,17,18]. Such repeated observations should be taken into consideration by the regulatory authorities. The current proposal for a new Tobacco Product Directive of the European Union [19,20], dictating 20 mg/mL maximum nicotine content in EC liquids, could potentially reduce the effectiveness of ECs as smoking substitutes; no beneficial effect of such a measure is expected, since the historically defined lethal nicotine dose has been recently challenged [21], while other toxic products (such as household cleaning products, bleach *etc*.) are available without restrictions in package size and content.

The most important reasons for participants to initiate ECs were to reduce or completely quit smoking and to reduce exposure of family members to second-hand smoking. It seems that these subjects are well-informed about the adverse health effects of smoking and are willing to try an alternative product which they consider less harmful. However, 11% of the subjects considered ECs as completely harmless. Studies have shown that low levels of toxic chemicals are released from EC use [22,23], while the effects of inhaling food-approved flavourings have not been adequately assessed [24,25]. Thus, we could expect some residual harm from EC use, which however seems to be much lower compared to smoking. Since it is still too early for follow-up studies that could define the long-term effects of EC use, proper education is needed so that smokers can make informed decisions about EC use.

More than half of the participants reported some side effects which they attributed to EC use. The most common were dry mouth and throat, which have been observed in previous studies [4,5,6]. They may be associated with the water-absorbing properties of propylene glycol and glycerol, which are the main constituents of EC liquids. However, these side effects were counteracted by significant benefits in physiologic functions. Additionally, a substantial proportion of participants reported pre-existing disease conditions, including respiratory disease, with benefits observed by the majority after initiating EC use. As expected, former smokers were more likely to report beneficial health effects compared to current smokers (often called dual users). The latter should be encouraged to completely eliminate tobacco cigarette use; a recent longitudinal study showed that 46% of dual users managed to quit smoking at 1 year follow-up [26].

Obviously, this survey cannot provide definite proof about the direct association between the experienced benefits and EC use. Moreover, as previously mentioned, the convenient sample of this survey precludes from generalizing the findings to the whole population; however, the potential of switching from smoking to ECs to favourably affect the prognosis of a variety of diseases needs to be studied further. In any case, benefit is expected to come from the reduction or complete cessation of smoking and not from any therapeutic effects of ECs *per se* [27].

A very small proportion of the population (0.5%) reported that they were not smokers before initiating EC use; there was evidence from responses to survey questionnaire that some of them were former smokers at the time of EC use initiation. More than half of them were using non-nicotine liquids. The reason for this is probably that they wanted to enjoy the experience and flavours without risking addiction to nicotine. Although this subgroup was a minor proportion of the survey population, it must be emphasized that ECs should be used by smokers only, as a substitute to smoking, and not as a new trend or habit since the long-term effects of use are currently unknown. Continuous monitoring of use by non-smokers or by youngsters is warranted. There is currently no evidence of adoption of EC use by such a population, with the CDC reporting that only 0.5% of non-smoking adolescents had tried EC in the past 30 days [28].

## 6. Conclusions

In conclusion, in this large sample of dedicated EC users, it seems that ECs are used as long-term substitutes to smoking. They can be effective even in subjects who are highly dependent on smoking and are heavy smokers. Mild temporary side-effects and significant benefits are reported by this population. Motivation for using ECs comes from their expected less harmful potential compared to smoking. The results should however be interpreted with caution considering the convenience sample of dedicated users usually participating in such surveys. More interventional and population studies are needed, which should take into consideration that every user has different preferences in terms of products choice, in order to further evaluate the effects of EC use at a population level.
